# Exploring the relationship between lower limb strength, strength asymmetries, and curvilinear sprint performance: Findings from a pilot study

**DOI:** 10.1177/00368504241247998

**Published:** 2024-04-23

**Authors:** Matic Sašek, Nejc Šarabon, Darjan Smajla

**Affiliations:** 1 Faculty of Health Sciences, 68960 University of Primorska, Izola, Slovenia; 2 Andrej Marušič Institute, 68960 University of Primorska, Koper, Slovenia; 3 Laboratory for Motor Control and Motor Behavior, S2P, Science To Practice, Ltd, Ljubljana, Slovenia; 4 Ludwig Boltzmann Institute for Rehabilitation Research, Vienna, Austria

**Keywords:** Curvilinear sprint, lower limb strength, strength asymmetries, deficit

## Abstract

Team sports involve various sprinting actions, including curvilinear sprints, yet their neuromuscular factors have been understudied. The aim of this cross-sectional study was to investigate the relationship between lower limb muscle strength, strength asymmetries, linear sprint and curvilinear sprint performance. At two visits 12 male (age: 24.8 ± 4.7 years, height: 1.82 ± 0.06 m, body mass: 80 ± 6.58 kg) and 6 female (age: 20.8 ± 1.33 years, body height: 1.60 ± 0.02 m, body mass: 55.3 ± 2.88 kg) student-athletes completed isometric strength measurements of the knee flexors (K_F_), knee extensors (K_E_), hip abductors (H_ABD_), hip adductors (H_ADD_), as well as linear sprint and curvilinear sprint to the right and left. Sprint split times over 30 m (t_30_) were measured and curvilinear sprint split time deficits (*t*_30deficit_) and inter-limb strength asymmetries were calculated. Very large negative correlations were observed between H_ADD_ and H_ABD_ strength on one side and *t*_30_ of curvilinear sprint to the left (*r* = −0.75 and −0.71; *p* < 0.001) and right (*ρ* = −0.81 and −0.70; *p* < 0.001) on the other. The regression model consisting of *H*_ADD_, *H*_ABD_, and *K*_F_ explained 76% and 67% of the variance in left and right curvilinear sprint *t*_30_, respectively. Similarly, 59% of the left curvilinear sprint *t*_30deficit_ variance was explained by the H_ABD_ and K_F_ strength. High inter-limb H_ABD_ strength symmetry was related to better left and right curvilinear sprint *t*_30_ (*r* = 0.71 and ρ = 0.75, *p* < 0.001). These results highlight the pivotal role of hip strength for curvilinear sprint speed, and emphasize the need of symmetrical H_ABD_ muscle strength to optimize neuromuscular function during curvilinear sprint.

## Introduction

The demands of ball game sports require different types of sprinting actions, such as linear sprint (LS), curvilinear sprint (CS), and change of direction (CoD) sprints.^1^ As a result, multidirectional speed, constitutes a fundamental pillar of athletic performance in disciplines such as football, basketball, and handball.^2–5^ Despite this significance, the research community and sports practitioners have primarily concentrated on investigating CoD and LS rather than CS. The desire to “develop a fast athlete” has drawn much attention to the factors that either constrain or augment sprint performance and muscle strength has been found to correlate with linear and CoD sprinting.^6,7^ Investigations have unveiled the significance of knee flexors (K_F_) and hip extensors strength for linear and also CoD speed,^8–10^ and high levels of hip adductor (H_ADD_) and abductor (H_ABD_) strength were found to play an important role for modified T-test and the 90° CoD sprint performance.^11,12^ Furthermore, the presence of inter-limb asymmetries in hip and knee strength has emerged as a crucial predictor of CoD sprint performance.^7,13^ These findings hold substantial implications and have prompted coaches to incorporate specific modes of strength exercises into training programmes that efficiently improve linear and CoD sprint performance^14,15^ and prevent injury^16^ of athletes. However, in this multidirectional speed-strength puzzle, the studies on CS are missing, leaving the relationship between muscle strength and CS performance inadequately understood.

The biomechanical properties of CS are in some ways different and at the same time similar to linear, and CoD sprints.^2^ Because sprints are completed with some degree of curvature in a cyclic but asymmetric mode,^17^ studies have shown specific changes in the function of the neuromuscular system when sprinting in a curve. In order to counteract centripetal forces, there is a distinct pattern of muscle activation and stride characteristics observed in the right and left limbs.^18–20^ Notably, lower limb muscles, responsible for generating joint moments in both medio-lateral and antero-posterior directions, appear to play a pivotal role in propelling the athlete during CS.^21^ Despite previous studies demonstrating a high positive correlation between lower limb strength and LS speed,^10^ the precise relationship between the strength of the K_F_, knee extensors (K_E_), H_ADD_, and H_ABD_ on one side, and CS performance on the other remains unknown. Furthermore, the strength asymmetries of these muscle groups could be an important factor in CS performance due to the asymmetric nature of CS execution.^22^ Because the CS is generally slower than linear sprint, the difference in their performance is expressed as the CS deficit. This variable has the potential to distinguish between the weaker and stronger sprint sides. It appears that it is associated with lower limb asymmetries and limb dominance rather than vertical jump performance.^23^ Therefore, delving into the relationship between lower limb strength, strength asymmetries, and CS deficit is also intriguing.

To piece together the multidirectional speed-strength puzzle, we aimed to investigate the relationship between lower limb muscle strength, strength asymmetries, linear, and CS sprint performance. The outcomes of this study will assist coaches in optimizing strength training by targeting muscle groups relevant to improving curvilinear speed. Additionally, the study will provide researchers with a foundation for further exploration into the essential neuromuscular aspects of CS performance. Our hypothesis poses a significant positive correlation between lower limb strength and performance in CS and LS, while expecting negative correlations in relation to strength asymmetries. Notably, the hip muscles are anticipated to exhibit stronger correlations with CS performance.

## Materials and methods

### Procedures

**Figure 1.**
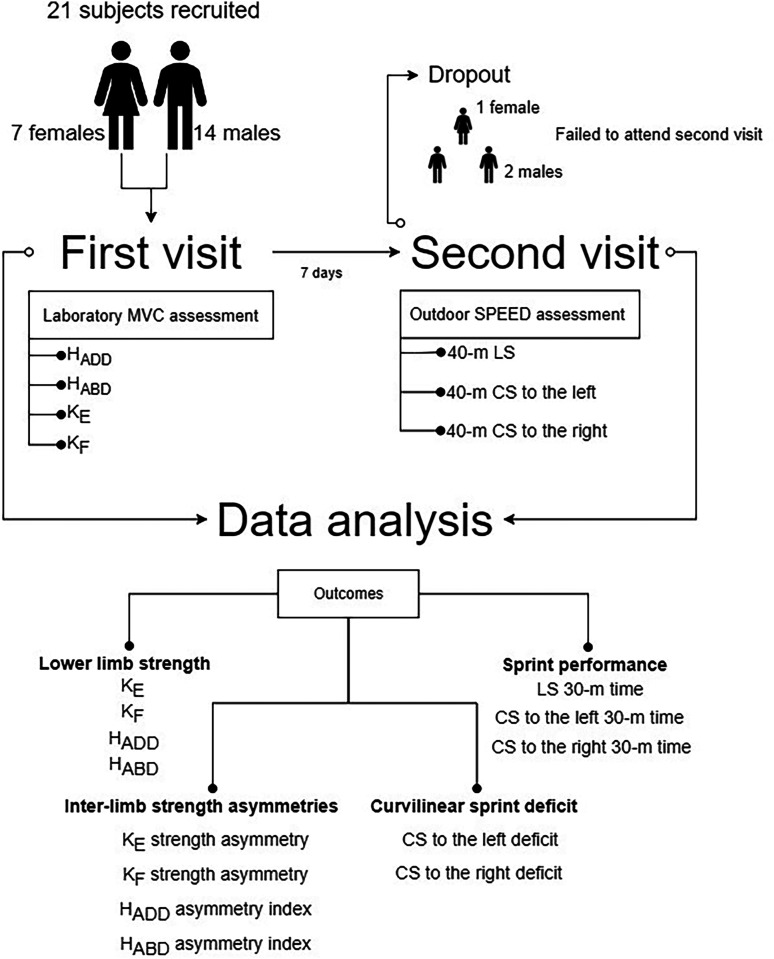
The figure illustrates the study design and recruitment process. Lower limb strength of hip adduction (H_ADD_), hip abduction (H_ABD_), knee extension (K_E_), and knee flexion (K_F_) was measured during the first visit, while LS and CS performances were assessed during the second visit as 30-m split times (t30). Among the 21 subjects recruited for the first visit, one female and two males dropped out of the analyses due to non-attendance at the second visit. LS: linear sprint; CS: curvilinear sprint.

For this study, a cross-sectional design with two visits separated by 7 days was employed. The procedures and participant recruitment process are illustrated in Figure 1. During the first visit to the laboratory, body mass and body height were measured using a body composition analyser TBF-300A (Tanita Corporation of America Inc., Arlington Heights, IL, USA) and stadiometer Seca 202 (Seca Ltd, Hamburg, Germany). Subsequently, the isometric strength of the K_F_, K_E_ H_ABD_, and H_ADD_ was assessed. During the second visit, the performance of the LS and CS to the right and left was measured on an artificial turf outdoor football field.

### Participants

Eighteen participants, comprising 12 males and 6 females, volunteered to participate in this study. The characteristics of the participants are detailed in Table 1. All participants were student-athletes who were still actively engaged in football, basketball, handball, and strength and conditioning training at least 5 times per week. Exclusion criteria included the presence of neurological or musculoskeletal disorders or lower leg injuries in the past 6 months. All participants signed a written informed consent form before the start of the study and were thoroughly informed about the experimental procedures. The study protocol complied with the principles of the Declaration of Helsinki and was approved by the Slovenian Medical Ethics Committee (approval number 0120-690/2017/8).

**Table 1.** Descriptive statistics for age, body height, body mass, strength, asymmetries in strength, and speed performance.

		Variable	Mean (±SD)
Subject's characteristics	Male (*n* = 12)	Age	24.80 (4.70)
		Body mass [kg]	80.00 (6.58)
		Body height [m]	1.82 (0.06)
		Sport experience years	8.00 (2.22)
	Female (*n* = 6)	Age	20.8 (1.33)
		Body mass [kg]	55.30 (2.88)
		Body height [m]	1.60 (0.02)
		Sport experience years	6.00 (1.55)
			
Lower limb strength [N m/kg]	Left knee extension		4.82 (0.90)
	Right knee extension		4.81 (0.87)
	Left knee flexion		2.09 (0.37)
	Right knee flexion		2.20 (0.38)
	Left hip abduction		2.10 (0.42)
	Right hip abduction		2.12 (0.41)
	Left hip adduction		2.15 (0.58)
	Right hip adduction		2.31 (0.50)
			
Sprint performance [s]	Linear sprint *t*_30_		4.62 (0.29)
	Left curvilinear sprint *t*_30_		5.00 (0.33)
	Right curvilinear sprint *t*_30_		5.02 (0.36)
			
Curvilinear sprint deficit [%]	Left curvilinear sprint *t*_30deficit_		8.09 (3.20)
	Right curvilinear sprint *t*_30deficit_		8.80 (4.96)

*t*_30_: time at 30 m distance; *t*_30deficit_: deficit in the time at 30 m distance.

#### Strength measurement

Before the measurement, participants underwent a standardized warm-up, which included 5 min of self-paced, low-intensity running, 8 dynamic stretching exercises, and 8 strengthening exercises. Next, the strength of H_ABD_ and H_ADD_ was bilaterally measured by performing maximal voluntary contraction (MVC) using the MuscleBoard® dynamometer (Polygon Group, Ltd, Zagreb, Croatia), a reliable device for assessing and monitoring hip muscle strength.^24^ Participants were seated in the device with their hips at a 45° flexion, knees fully extended and locked, ankles in slight dorsiflexion, and hands placed behind the device for support. Stabilization was ensured with a non-elastic strap across the anterior side of the pelvis (for visual reference, readers are encouraged to see Marušič et al.^24^). To assess K_F_ and K_E_ strength, unilateral MVCs were performed on a specialized knee dynamometer (S2P Ltd, Ljubljana, Slovenia) with integrated force sensors (Z6FC3-200 kg and PW10AC3-200 kg, respectively, HBM, Darmstadt, Germany).^25^ The dynamometer's seat and distal shin pad were individually adjusted to allow a seated position with 90° angle at the hip and 60° at the knee (0° is considered full knee extension). The medial femoral epicondyle served as the reference for the participant's knee axis of rotation, aligned with the mechanical axis of the dynamometer. Fixation was ensured by non-elastic straps tightened across the anterior side of the pelvis, just above the knee, and 3–5 cm proximal to the medial malleolus.^26^ All tests were performed in a randomized order and repeated three times with 60-s rest between trials.^27^ Participants were instructed to push as hard as they could against the support for 3 and were verbally encouraged during all trials.^28^

#### Linear and CS measurement

When testing sprint performance all participants wore football boots. Prior to the testing procedure, the warm-up from the first visit was repeated, and three submaximal LS and CS were added at the end. Next, participants performed three 40-m LS and CS to the right and the left in a balanced and random order. The split times of the sprints were measured every 5 m with electronic single-beam timing gates (Brower Timing Systems, Utah, USA). The sensors were placed at hip height. For the CS to the right and CS to the left, the circumference of the centre of the football field with a radius of 9.15 m was used.^29^ The sprint start was always 0.5 m behind the first pair of timing gates, directly behind the horizontal lines of the football field. Between sprints, at least 5-min rest was used. The 40-m distance allowed the athletes to reach their maximal sprinting speed, covering most, if not the entire, acceleration phase of the sprint. At the time of testing, ambient conditions ranged from 18 to 22 °C air temperature, 65% to 80% humidity, and a wind speed of 1.7–3.6 m/s.

#### Strength, strength asymmetries, sprint performance and CS deficit

During the MVC strength tests, the torque signals for the left and right limbs were sampled and processed automatically using custom developed software (ARS Dynamometry, S2P, Ljubljana; developed in Labview 20.0f1, National Instruments, Austin, Texas, USA). Maximal torque values were normalized to body mass [N m/kg] and the best trials were selected as lower limb strength indicators. Inter-limb asymmetries were then calculated for the unilaterally performed strength tests (K_F_ and K_E_) according to Equation 1, while inter-limb asymmetry indexes were calculated for the bilaterally performed strength tests (H_ABD_ and H_ADD_) according to Equation 2.^30^

(1) $$\mathrm{Inter}\text{-}\mathrm{limb}\;\mathrm{strength}\;\mathrm{asymmetry}\;(\text{\%})=\frac{\mathrm{stronger}\;\mathrm{limb}\text{-}\mathrm{weaker}\;\mathrm{limb}}{\mathrm{stronger}\;\mathrm{limb}}\;\times 100$$

(2) $$\mathrm{Inter}\text{-}\mathrm{limb}\;\mathrm{asymmetry}\;\mathrm{index}\;(\text{\%})=\frac{\mathrm{stronger}\;\mathrm{limb}\text{-}\mathrm{weaker}\;\mathrm{limb}}{\mathrm{stronger}\;\mathrm{limb}+\mathrm{weaker}\;\mathrm{limb}}\;\times 100$$

The split times at 30 m (*t*_30_) were determined for LS and CS with an accuracy of 0.01 s, and the fastest trials were selected to determine sprint performance. Finally, the CS *t*_30_ was subtracted from the LS times and expressed as a percentage to obtain the right and left curvilinear time deficit (*t*_30deficit_).^31^

### Statistical analyses

Analyses were conducted using SPSS version 26.0 (SPSS, Chicago, IL). Descriptive data are presented as the mean and standard deviation (SD). The normality of the distribution was assessed using the Shapiro-Wilk test. The correlations between strength, asymmetries, and sprints performance were calculated using Pearson's *r* correlation coefficient. In the case of a non-normal distribution, Spearman's test was used. The magnitude of the correlation was interpreted as trivial (< ± 0.1), small (± 0.1–0.3), moderate (± 0.3–0.5), large (± 0.5–0.7), very large (± 0.7–0.9), or nearly perfect (> ± 0.9).^32^ Multiple linear stepwise regressions were conducted, with *t*_30_ (of LS, CS to the right, and CS to the left) and *t*_30deficit_ (reflecting CS to the right and CS to the left) serving as the dependent variables. The predictor variables included all strength or all strength asymmetry parameters in two sets of regressions. Predictors were added to the model if they demonstrated statistical significance (*p* < 0.05) in contributing to the explained variance in *t*_30_ and *t*_30deficit_. To ensure the robustness of our analysis, we conducted Durbin-Watson statistics and diagnostic tests for collinearity. We established conservative thresholds with tolerance values of ≥ 0.3 and variance inflation factors of ≤ 3.^33^ The significance level was set at *p* < 0.05. Due to multiple correlations performed we applied the Bonferroni-Holm correction to adjust the *p*-values obtained from the correlation analyses.^34^

## Results

**Figure 2.**
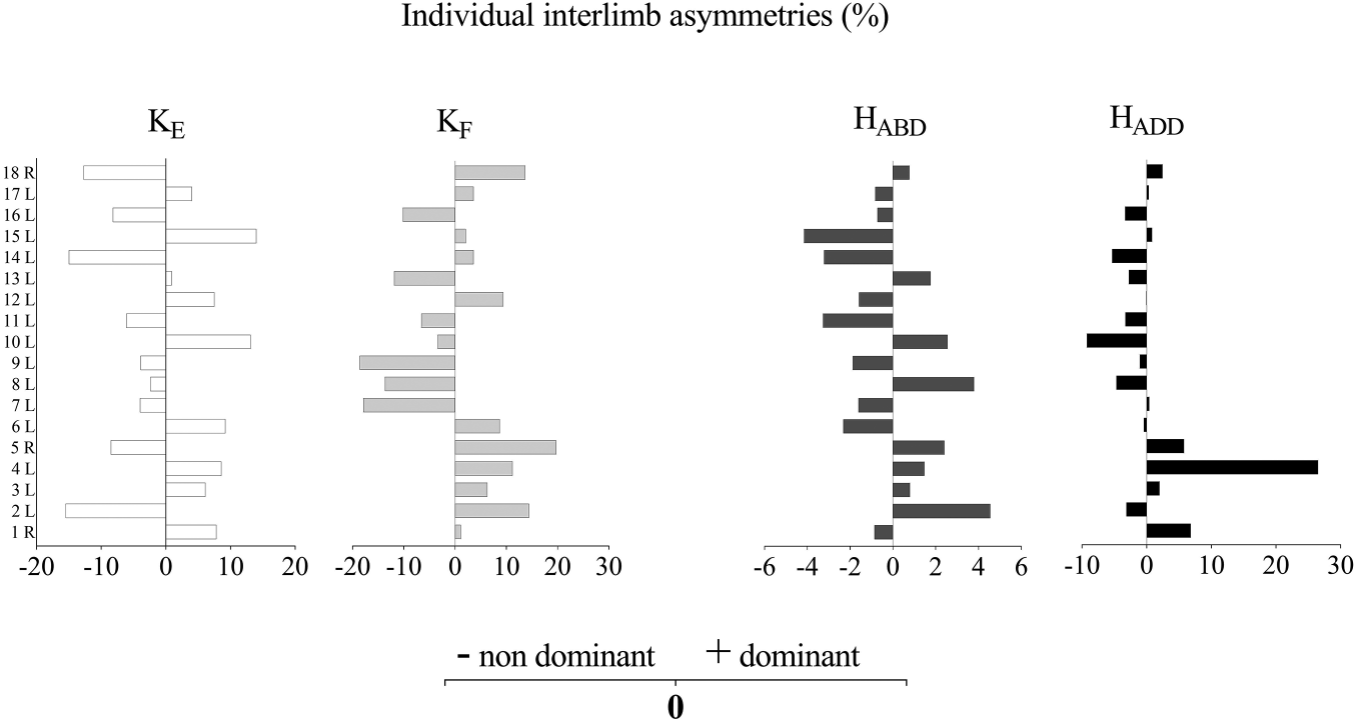
Individual inter-limb strength asymmetry and index for knee extension (K_E_), knee flexion (K_F_), hip abduction (H_ABD_), and hip adduction (H_ADD_) peak torque.

*Note*. On the *y*-axis, the identification number of the individual and the dominant limb (referred to the push-off leg) are shown. The *x*-axis shows inter-limb strength asymmetries and indexes in percent. Negative values reflect the asymmetries towards non-dominant limb, while positive values reflect the asymmetries towards dominant limb.

Table 1 presents descriptive statistics for the strength and sprint performance variables. The individual strength asymmetries are presented in Figure 2. The Shapiro-Wilk test revealed non-normal distribution for the right limb K_F_ strength, H_ADD_ strength asymmetry index, LS *t*_30_, CS *t*_30_ to the right, and *t*_30deficit_ of CS to the right (all *p* < 0.05). The mean inter-limb asymmetries of K_E_ (8.19 ± 4.40%) and K_F_ (9.78 ± 5.82%) were greater than those of H_ABD_ (2.14 ± 1.23%) and H_ADD_ (4.37 ± 6.98%).

### Association between strength and sprint performance

The correlations between strength and sprint performance are shown in Table 2. After applying the Bonferroni-Holm correction, the H_ADD_ strength of the left and right legs showed significant negative correlation with *t*_30_ of LS (ρ = −0.72 and −0.72, respectively) and CS to the right (ρ = −0.76 and −0.81, respectively). Additionally, significant negative correlations were observed between right H_ADD_ and H_ABD_ strength and *t*_30_ of CS to the left (*r* = −0.75 and −0.71, respectively). Right H_ABD_ also showed a significant negative correlation with *t*_30_ of CS to the right (ρ = −0.70).

**Table 2.** Correlation coefficients between knee and hip strength, sprint performance and curvilinear sprint deficit.

	Knee strength				Hip strength			
	Extension		Flexion		Abduction		Adduction	
Sprint variable	Left	Right	Left	Right	Left	Right	Left	Right
Linear *t*_30_	−0.56^s^	−0.57^s^	−0.58^s^	−0.61^s^	−0.32^s^	−0.41^s^	−0.72^s^*	−0.72^s^*
Left curvilinear *t*_30_	−0.62	−0.55	−0.56	−0.68^s^	−0.67	−0.71*	−0.68	−0.75*
Left curvilinear *t*_30deficit_	−0.09	0.09	−0.04	−0.07^s^	−0.66	−0.62	−0.07	−0.19
Right curvilinear *t*_30_	−0.63^s^	−0.42^s^	−0.60^s^	−0.56^s^	−0.65^s^	−0.70^s^*	−0.76^s^*	−0.81^s^*
Right curvilinear *t*_30deficit_	−0.28^s^	−0.08^s^	−0.28^s^	−0.19^s^	−0.62^s^	−0.57^s^	−0.21^s^	−0.31^s^

*t*_30_: time at 30 m distance; *t*_30deficit_: deficit in the time at 30 m distance.

*p < 0.00147

s Spearman rho (ρ) correlation coefficient.

### Association between strength asymmetry and sprint performance

Table 3 shows the correlation coefficients between strength asymmetries, sprint performance, and CS deficit. Only the H_ABD_ strength asymmetry index showed significant very large correlations with left and right CS *t*_30_ (*r* = 0.71 and ρ = 0.75, respectively).

**Table 3.** Correlation coefficients between inter-limb strength asymmetries, sprint performance, and curvilinear sprint deficit.

	Strength asymmetry		Asymmetry index	
Sprint variable	Knee extension	Knee flexion	Hip abduction	Hip adduction
Linear *t*_30_	0.40^s^	−0.05^s^	0.62^s^	0.50^s^
Left curvilinear *t*_30_	0.38	−0.11	0.71*	0.35^s^
Left curvilinear *t*_30deficit_	0.10	−0.13	0.28	−0.34^s^
Right curvilinear *t*_30_	0.26^s^	0.09^s^	0.75^s^*	0.27^s^
Right curvilinear *t*_30deficit_	−0.12^s^	0.13^s^	0.37^s^	−0.36^s^

*t*_30_: time at 30 m distance; *t*_30deficit_: deficit in the time at 30 m distance.

*p < 0.0026.

s Spearman rho (ρ) correlation coefficient.

### Regression analyses

No multicollinearity was detected in the regression models (variance inflation factors < 1.93; tolerance > 0.70) presented in Table 4. Knee and hip strength variables explained 61%, 76%, and 67% of the variance in *t*_30_ of LS, CS to the left, and CS to the right, respectively. For the LS and CS to the left and right, H_ADD_ and K_F_ strength were included. Additionally, in the model explaining the performance of CS to the left, H_ABD_ strength was present. The model consisting of H_ABD_ and K_F_ strength explained 59% of the variance in *t*_30deficit_ of CS to the left.

**Table 4.** Strength and strength asymmetry predictors of linear and curvilinear sprint performance variables.

Dependent sprint variable	Predictors	*B*	SE	*ß*
	*Strength predictors*			
Linear *t*_30_	Constant	5.961	0.289	
	H_ADD_ right	−0.274	0.111	−0.472
	K_F_ right	−0.321	0.147	−0.417
Left curvilinear *t*_30_	Constant	6.81	0.287	
	H_ADD_ right	−0.218	0.118	−0.335
	K_F_ right	−0.315	0.134	−0.365
	H_ABD_ right	−0.292	0.131	−0.368
Right curvilinear *t*_30_	Constant	6.756	0.331	
	H_ADD_ right	−0.398	0.127	−0.548
	K_F_ right	−0.371	0.169	−0.385
Left curvilinear *t*_30deficit_	Constant	13.92	3.402	
	H_ABD_ left	−6.348	1.381	−0.831
	K_F_ right	1.562	0.669	0.422
			
	*Strength asymmetries predictors*			
Linear *t*_30_	Constant	4.318	0.115	
	H_ABD_ symmetry index	0.143	0.047	0.605
Left curvilinear *t*_30_	Constant	4.593	0.115	
	H_ABD_ symmetry index	0.188	0.047	0.710
Right curvilinear *t*_30_	Constant	4.521	0.112	
	H_ABD_ symmetry index	0.233	0.046	0.787

*ß*: standardized coefficient; *B*: unstandardized coefficient; H_ADD_: hip adduction; H_ABD_: hip abduction; K_F_: knee flexion; SE: standard error; *t*_30_: time at 30 m distance; *t*_30deficit_: deficit in the time at 30 m distance.

Into the sets of regressions constructed of strength asymmetry variables only, H_ABD_ strength asymmetry was the only predictor of LS, CS to the left, and CS to the right *t*_30_, explaining 32%, 50%, and 62% of their variance, respectively.

## Discussion

The primary objective of this study was to investigate the relationships between lower limb muscle strength, strength asymmetries, CS performance, and CS deficit. Our findings indicate that high H_ABD_ and H_ADD_ strength exhibit very large correlations with enhanced LS and CS performance. In combination with the K_F_ strength these muscle groups explained 61%, 76%, 67%, and 59% of the variance in LS, CS times, and deficit. We observed that diminished LS and CS performance is linked to inter-limb asymmetries in H_ABD_ strength. As a single predictor, the H_ABD_ strength asymmetry index explained 50% and 62% of the variance in CS times. Overall, the outcomes of our study suggest that higher levels of knee and hip muscle strength and symmetry in strength are crucial for rapid curvilinear sprinting.

### Relationship between lower limb strength and linear sprint performance

Our study findings corroborate previous research, providing further evidence of a strong correlation between the muscular strength of key sprint propulsors and LS speed.^10,35,36^ Notably, our regression model emphasizes the prominent role of H_ADD_ and K_F_ strength for LS performance, as evidenced by nonsignificant but still large to very large positive correlations observed with LS *t*_30_. This underscores the significance of hamstring muscle size in facilitating linear sprint performance,^37^ and it's substantial contribution to forward propulsion during sprinting.^8,38–41^ In contrast, a noteworthy gap exists in the current literature concerning the relationship between H_ADD_ strength and linear sprinting performance. Our study misaligns with the findings of Królikowska et al.,^12^ suggesting a less pronounced correlation with 20-m LS time, based on the relatively trivial relationship (*r* = 0.17 and 0.06). However, the very large correlations between H_ADD_ and *t*_30_ of LS (ρ = −0.72) observed in our study could be attributed to the longer distance used as a sprint performance indicator. These may find theoretical support in the role of the hip muscles in sagittal plane stabilization, indirectly enabling increased production of vertical and horizontal ground reaction forces during sprinting.^42^ Thus, greater H_ADD_ strength may lead to improved sprinting technique and more optimal sagittal movement mechanics when sprinting straight.^43^ In line with this premise, we also anticipated strong correlations between H_ABD_ strength and LS *t*_30_. However, our findings did not yield statistically significant correlations in this regard. It is important to acknowledge that our study's methodology does not establish causality, thus warranting further research to distinguish the contribution of H_ADD_ and H_ABD_ strength to LS mechanics.

### Relationship between lower limb strength and CS performance

Prior observations, which have documented high electromyographic activity of the m. gluteus medius during the stance phase of the outside leg in curvilinear sprinting, suggest their pivotal role as primary propulsors during CS.^2^ This is a distinction to their role in LS,^18^ which becomes apparent in our study by observing a notably stronger correlation between H_ABD_ strength and CS to the left and to the right times (*r* = −0.71 and ρ = −0.70) compared to the correlation between H_ABD_ and LS time (ρ = −0.32 and −0.41). In addition to H_ABD_, K_F_ and H_ADD_ were also included in the regression models for predicting CS and LS performance, a result that aligns with our expectations as these muscle groups contribute to propulsion in both types of sprints.^21^

On the other hand, evidence on neuromuscular factors influencing CS deficit is lacking. Loturco et al.^23^ have demonstrated a trivial correlation between the squat jump height and CS deficit, however, no study has assessed its correlation with muscle strength. Although statistically nonsignificant, our results elucidate four times larger correlations between H_ABD_ strength and *t*_30deficit_ of CS to the left and right compared to other muscle groups. This observation underscores the vital significance of H_ABD_ strength as a neuromuscular adaptation tailored to the distinct biomechanical prerequisites of curvilinear sprinting. Specific strength adaptations might interfere with smaller CS deficits of football players compared to sprinters, highlighting the influence of the athlete's sport and reaffirming the sport-specific nature of adaptations.^21,44^

### Relationship between strength asymmetries and CS performance

Although CS is characterized as an asymmetric cyclic motor task,^45^ the relationship between strength asymmetries in relevant lower limb muscles and its performance remains unexplored. Previous research has illuminated the significant relationship between CoD speed and inter-limb strength asymmetries.^7,13^ Coratella et al.^7^ reported strong positive correlations between inter-limb asymmetries in isokinetic concentric strength of K_F_ and CoD time, while Ujaković and Šarabon^13^ highlighted the pivotal role of inter-limb asymmetries in rate of torque development of H_ABD_ in T-test performance. Our study revealed a significant relationship between H_ABD_ strength asymmetry index and CS *t*_30_ (*r* = 0.71 and ρ = 0.75) which is evidence that the inter-limb balance in H_ABD_ strength is particularly important for CS performance. When sprinting in a curve to the right or left, electromyographic activity of the H_ABD_ muscles of the outside leg has been found to be lower than of the inside leg.^21^ Still, the inside leg H_ABD_ muscles must counteract adduction and external rotation of the femur, thereby stabilizing the hip.^2^ The symmetrical inter-limb strength of H_ABD_ could purposely contribute to a more coordinated neuromuscular function during strides, creating optimal biomechanical conditions for efficient generation of ground reaction forces, ultimately resulting in enhanced propulsion during CS. We must emphasize that the H_ABD_ strength asymmetries in our study ranged from a minimum of 0.72 to a maximum of 4.55%, reflecting low asymmetries. We infer that this is due to the H_ABD_ strength test procedure (i.e. performing MVC test bilaterally). Therefore, our conclusion regarding H_ABD_ strength asymmetries and CS performance should be considered with caution, and further evidence is needed to confirm our observations.

The relationship between lower limb strength and sprint performance indicated that an effective strategy to enhance multidirectional sprint performance would involve improving K_F_, H_ADD_, and H_ABD_ strength while concurrently reducing H_ABD_ inter-limb strength asymmetries. In addition to the commonly used strength exercises in training protocols for sprinting speed enhancement, coaches should consider incorporating unilateral exercises that involve high medio-lateral hip joint moments. Such exercises may contribute to increased hip muscle strength and potentially address strength asymmetry by emphasizing a larger volume of training on the non-dominant limb.^46^ To evaluate the effects of adjusted training protocols on CS performance, interventions focusing solely on increasing H_ADD_ and H_ABD_ strength, reducing hip strength asymmetry, or a combination of both approaches would be interesting. Furthermore, future research should explore the role of hip strength and strength asymmetries in the mechanics of CS to elucidate potential mechanisms underlying the high correlations observed in our study.

Regardless of the promising results of the present study, its limitations should be considered when interpreting the results. First of all, we must emphasize that the correlation analyses do not imply causality, thus future studies assessing the influence of hip strength training on CS performance should be conducted. It is also worth noting that we failed to target the strength of ankle muscles, multi-joint strength, and dynamic strength, which could potentially play a significant role in CS performance.^2^ And lastly, required sample size to obtain desirable statistical power (i.e. 0.8) was not a priori calculated thus this pilot investigation necessitates a cautious interpretation of the results.

## Conclusion

In summary, this study is the first of its kind to examine the correlations between lower limb muscle strength, strength asymmetries, and CS performance. The findings underline the pivotal role of K_F_, H_ADD,_ and H_ABD_ strength in CS performance. In addition to K_F_ and H_ADD_ playing an important role in LS performance, strong H_ABD_ muscles are associated with reduced CS time and diminished CS deficit. Furthermore, higher H_ABD_ strength asymmetry is an important predictor of CS performance. Coaches and practitioners are encouraged to prioritize H_ABD_ strengthening exercises, particularly when aiming to enhance CS speed and mitigate CS deficit. In future studies, researchers should investigate the effectiveness of such training regimes in developing CS performance and explore the role of hip strength and strength asymmetries in CS mechanics.
